# Predicting Ki-67 expression levels in breast cancer using radiomics-based approaches on digital breast tomosynthesis and ultrasound

**DOI:** 10.3389/fonc.2024.1403522

**Published:** 2024-07-11

**Authors:** Jie Liu, Caiying Yan, Chenlu Liu, Yanxiao Wang, Qian Chen, Ying Chen, Jianfeng Guo, Shuangqing Chen

**Affiliations:** ^1^ Department of Radiology, Nanjing Medical University Affiliated Suzhou Hospital, Suzhou, China; ^2^ Department of Ultrasound, Sir Run Run Hospital Nanjing Medical University, Nanjing, China; ^3^ Department of Ultrasound, Nanjing Medical University Affiliated Suzhou Hospital, Suzhou, China

**Keywords:** breast cancer, digital breast tomosynthesis, Ki-67 level, radiomics, ultrasound

## Abstract

**Purpose:**

To construct and validate radiomics models that utilize ultrasound (US) and digital breast tomosynthesis (DBT) images independently and in combination to non-invasively predict the Ki-67 status in breast cancer.

**Materials and methods:**

149 breast cancer women who underwent DBT and US scans were retrospectively enrolled from June 2018 to August 2023 in total. Radiomics features were acquired from both the DBT and US images, then selected and reduced in dimensionality using several screening approaches. Establish radiomics models based on DBT, and US separately and combined. The area under the receiver operating characteristic curve (AUC), accuracy, specificity, and sensitivity were utilized to validate the predictive ability of the models. The decision curve analysis (DCA) was used to evaluate the clinical applicability of the models. The output of the classifier with the best AUC performance was converted into Rad-score and was regarded as Rad-Score model. A nomogram was constructed using the logistic regression method, integrating the Rad-Score and clinical factors. The model’s stability was assessed through AUC, calibration curves, and DCA.

**Results:**

Support vector machine (SVM), logistic regression (LR), and random forest (RF) were trained to establish radiomics models with the selected features, with SVM showing optimal results. The AUC values for three models (US_SVM, DBT_SVM, and merge_SVM) were 0.668, 0.704, and 0.800 respectively. The DeLong test indicated a notable disparity in the area under the curve (AUC) between merge_SVM and US_SVM (*p* = 0.048), while there was no substantial variability between merge_SVM and DBT_SVM (*p* = 0.149). The DCA curve indicates that merge_SVM is superior to unimodal models in predicting high Ki-67 level, showing more clinical values. The nomogram integrating Rad-Score with tumor size obtained the better performance in test set (AUC: 0.818) and had more clinical net.

**Conclusion:**

The fusion radiomics model performed better in predicting the Ki-67 expression level of breast carcinoma, but the gain effect is limited; thus, DBT is preferred as a preoperative diagnosis mode when resources are limited. Nomogram offers predictive advantages over other methods and can be a valuable tool for predicting Ki-67 levels in BC.

## 1 Introduction

Breast Cancer (BC) is a malignant tumor with the greatest morbidity and mortality rate all around the world, which is a severe risk to women’s health (1). Ki-67 is a nuclear antigen that is closely connected with the invasiveness and proliferative activity of breast cancer (2). It serves as a significant marker for breast cancer category (3), prognosis, as well as predicting the effectiveness of preoperative neoadjuvant chemotherapy and endocrine therapy (4, 5). Currently, Ki-67 is mainly detected by immunohistochemistry (IHC), which requires tissue specimens to be obtained by core-needle biopsy or surgery. However, these procedures are invasive, time-consuming, not repeatable, and the limited number of samples obtained cannot thoroughly represent the tumor’s heterogeneity (6). Moreover, Ki-67 expression levels can change dynamically during the course of treatment (7), and IHC cannot be used as a routine means of dynamic monitoring. Therefore, it is crucial to find a non-invasive and accurate technique to assess Ki-67 expression before surgery.

During mammography, dense and inhomogeneous mammary glands might cause normal breast tissue to overlap with lesions, leading to decreased sensitivity and specificity in detection (8, 9). Digital breast tomosynthesis (DBT) is an advanced digital mammography technique that utilizes three-dimensional imaging technology to reduce breast tissue overlap, improving lesion visibility (10, 11). It provides increased sensitivity and specificity compared to traditional mammography, enabling the identification of initial, low-grade breast cancer (12). It is gradually becoming the current standard for breast screening and diagnosis.

Radiomics uses advanced data analysis techniques to assess biological indicators of breast cancer non-invasively before surgery (13), it offers significant potential in distinguishing between benign and malignant breast lesions, categorizing and grading breast cancer, and forecasting treatment response and risk of recurrence (14, 15), it also has major potential in evaluating the tumor microenvironment (16). Most current radiomics research to predict high Ki-67 expression relies on single-mode imaging or Magnetic Resonance Imaging (MRI). Nevertheless, the high cost, long examination time, and limited availability have impeded its practical application. Women diagnosed with breast cancer are more inclined to have DBT and US due to their efficiency, affordability, and ease of operation. A recent meta-analysis indicated that combining DBT and US can enhance the diagnosis accuracy of dense breasts (17), potentially serving as an alternative to MRI. Furthermore, a few studies have demonstrated that radiomics based on DBT and US is feasible and repeatable (18), and it has the potential to facilitate precision medicine.

**Figure 1:**
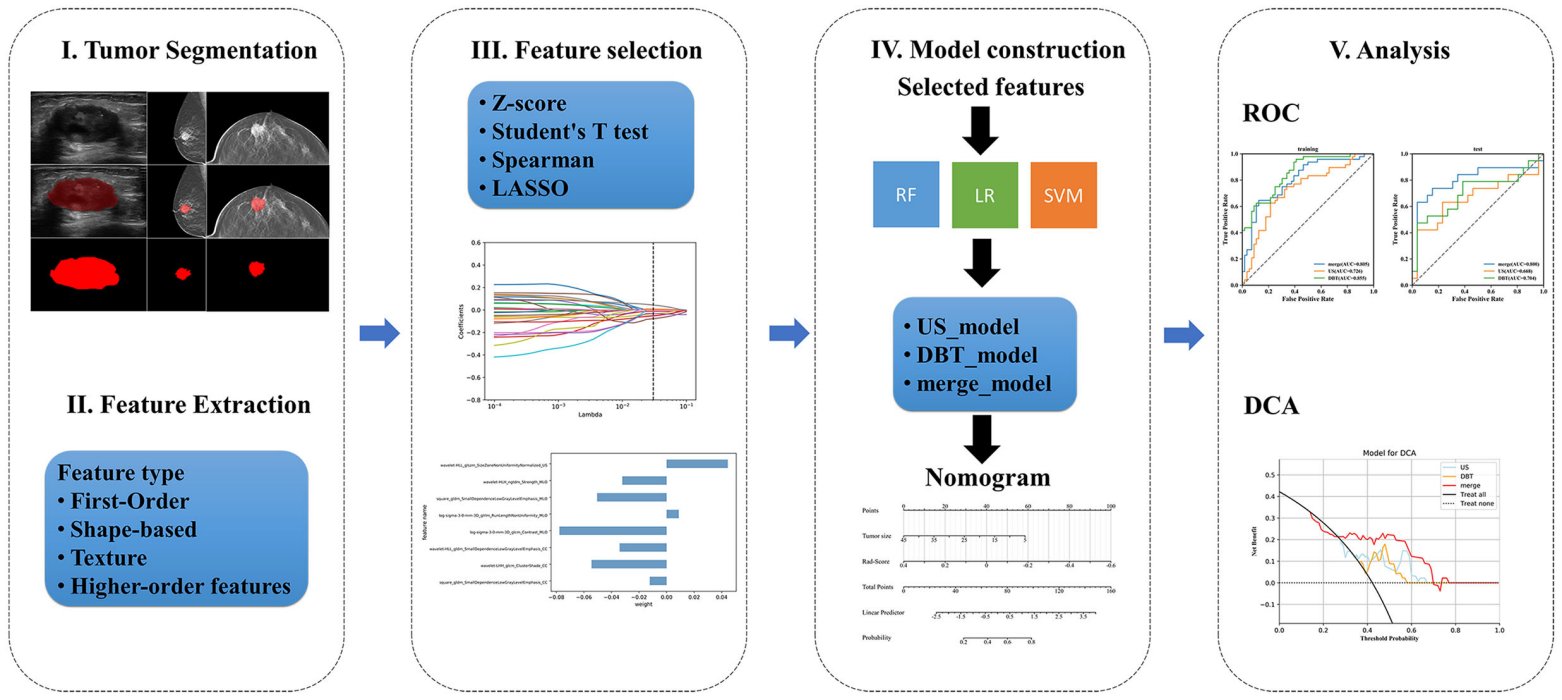
Workflow of predicting the Ki-67 level based on radiomics.

Therefore, the present study attempted to construct DBT, US, and fusion models utilizing the quantitative radiomics features extracted from DBT and US images, and to investigate whether the three models could enhance the diagnostic efficacy of preoperative noninvasive prediction for Ki-67 status. **Figure 1** displays the workflow.

## 2 Materials and methods

### 2.1 Patient selection

This retrospective research received approval from the Ethics Committee of the Suzhou Municipal Hospital (No. 2024320), and the need for written informed permission was waived. Patients pathologically diagnosed with breast tumors in the breast cancer screening center from June 2018 to August 2023 were enrolled.

The inclusion standards were (a) patients diagnosed with breast cancer via biopsy or surgical pathology; (b) DBT and US examinations were conducted within 1 week before surgery; (c) immunohistochemical indexes such as Ki-67 were perfected after operation.

The exclusion standards were (a) inadequate quality of DBT and US images for radiomics analysis owing to artifact, calcification, cystic degeneration, etc.; (b) patients had received any form of treatment (including neoadjuvant chemotherapy, radiotherapy, hormone therapy, surgery, and core-needle biopsy) before DBT and US examination; (c) lesions larger than 50 mm (not completely shown on a single plane); (d) patients with other primary tumors.

### 2.2 Clinical and pathological assessment

Clinical data including age, mass location, and menstrual status were acquired from the hospitalization system of our center, tumor size, calcifications, and burr edges were evaluated from medical images. ER, PR, HER2, and Ki-67 were detected by IHC of the surgical specimens, conducted by two senior attending physicians in the Department of Pathology. ER and PR status were taken for positive when ≥1% of tumor cell nucleus showed ER and PR staining. The diagnostic criterion for positive expression of Ki-67 was the percentage of tumor nuclei in the field of view of the hot spot in the section. According to the expert consensus of the 2020 International Breast Cancer Ki-67 Working Group (19), a cut-off value of 30% was used to classify high and low Ki-67 expression (≤30% as low expression and >30% as high expression). HER2 expression levels of 0 and 1+ were classified as negative, 3+ as positive, and 2+ required additional FISH testing: amplification was deemed positive, while non-amplification was deemed negative.

### 2.3 Image acquisition

All DBT images were obtained using a Selenia Dimensions system (Hologic, Bedford, MA, USA) on both the craniocaudal (CC) and mediolateral oblique (MLO) views. The DBT volumes were rebuilt with a slice interval of 1 mm and an in-plane pixel size of roughly 100 μm using the filtered back-projection reconstruction method. The scanning angle was 15° ± 7.5°. The US inspections were conducted by two sonographers with over 10 years of experience in breast diagnosis for each patient. Each patient was placed in the supine position, and the Resona R7 or Resona R9 ultrasonic diagnostic apparatus (Mindray, Shenzhen, China) was utilized, with the probe model L14-5WU. The probe frequency was set at 7-10 MHz, and the measurement standard for lesions was based on the 2013 American Co1lege of Radiology (ACR) Breast Imaging Reporting and Data System (BI-RADS) (20), the multifocal mass selected the largest mass for measurement, the gray-scale ultrasound imaging of the largest plane of each breast lesion was selected in all patients. Subsequently, all collected images were stored in digital imaging and communications in medicine (DICOM) format.

### 2.4 ROIs segmentation

The Regions of Interest (ROIs) were manually drawn layer by layer along the edge of the lumps by two imaging physicians with over 5 and 15 years of experience in breast diagnosis utilizing an open-source imaging platform ITK-SNAP (version 4.0.1, http://www.itk-snap.org), avoiding cystic and necrotic areas as much as possible. Subsequently, 40 image cases were chosen randomly for a consistency assessment.

### 2.5 Feature extraction

All images were standardized and normalized to reduce variability caused by different machines, with the intensities of the images adjusted to a range of 0-1. Then the open-source pyRadiomics package of Python (version 3.7.6) was used to extract the radiomics features from the CC, MLO images of DBT, and US images respectively. These features mainly encompass first-order features, shape features, texture features, and higher-order texture features (features extracted through transformations such as wavelet and Laplace filter (LoG)). The specific parameters can be referred to https://pyradiomics.readthedocs.io/. Overall, 1427 features were separately acquired from the CC and MLO views of DBT images, whereas 1239 features were obtained from the US images. All features were merged as features of the fusion model.

### 2.6 Feature selection

**Figure 2:**
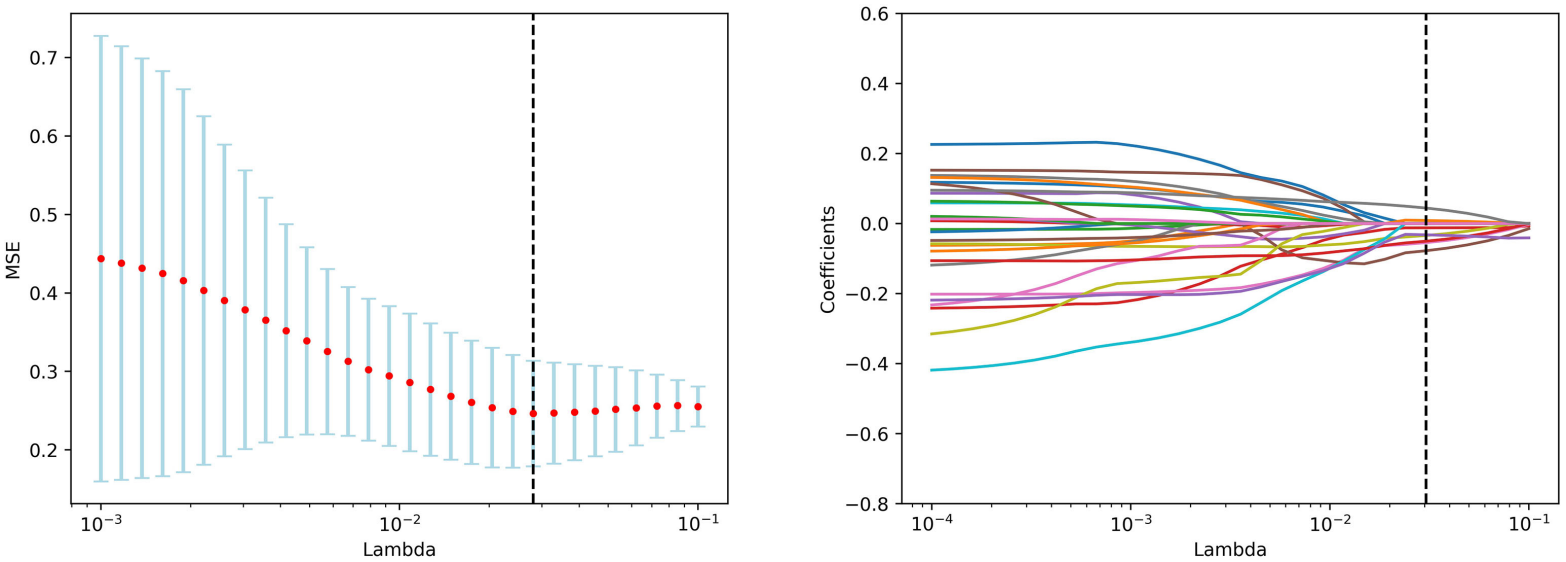
LASSO algorithm for radiomics features selection in the training set.

The participants were divided into the training and test sets randomly with a ratio of 7:3. The feature screening procedure was applied in the training set to screen the optimal radiomics features in 4 steps. Firstly, the extracted features were standardized by z-score normalization in both the training and the test set separately to provide a uniform standard of feature values and enhance comparability between features, achieving proportional scaling of the original data. Secondly, The Student’s T test was performed on all radiomics features and only features with a *p*-value <0.05 were considered potentially predictive and retained. Thirdly, RF was used to rank features from high to low. Then, the Spearman correlation coefficient was used to examine the relevance between features. Features with a coefficient > 0.9 were considered highly associated, and the one with a lower RF score was discarded. Finally, The Least Absolute Shrinkage and Selection Operator Method (LASSO) was performed for feature dimensionality reduction to select features further, the optimal tuning parameter λ was selected using 10-fold cross-validation, and features with non-zero regression coefficients were selected from these candidate features (**Figure 2**).

### 2.7 Model construction

Radiomics models: The features of the three models were input into three commonly used machine learning methods separately after the LASSO algorithm: Random Forest (RF), Logistic Regression (LR), Support Vector Machine (SVM), and the three prediction models (DBT_, US_, and merge_) were constructed using the “scikit-learn” package in Python (version 3.7.6). The predictive performance of the 9 models was presented in a table, and the optimal model was chosen by comparing the AUC values of the training and test sets. For the optimal model selected, the ROC curves of the training and validation sets were summarized to compare the differences in the AUC values of DBT, US, and fusion models. The Decision Curve Analysis (DCA) was used to quantify the net gains at different threshold probabilities in the validation set to evaluate the clinical utility of the three models.

Nomogram model: The output of the classifier with the best AUC value was converted into Rad-Score and was regarded as a Rad-Score model, and then we utilize the univariate and multivariate logistic regression to find the best clinical predictors, *p* < 0.05 were considered as the risk factors. Subsequently, the Rad-Score and risk predictors were integrated to construct the nomogram model. The model’s stability was evaluated using AUC, calibration curve, and DCA.

### 2.8 Statistical analysis

Statistical analyses of the data were conducted using the “SciPy. stats” package of Python (version 3.7.6) and graphing were performed using the “matplotlib. pyplot” package. The AUC, 95% confidence interval (95% CI), accuracy, sensitivity, and specificity were utilized to validate the predictive performance of the models. DeLong test was applied to evaluate the differences in AUC values between the models, *p* < 0.05 (two-tailed) was considered statistically significant.

## 3 Results

### 3.1 Clinical characteristics

A total of 149 cases were finally enrolled, consisting of 82 individuals with low Ki-67 level and 67 with high Ki-67 level. All participants were female, aged 30 to 86 years old, with an average of 59.01 ± 11.78 years old. The patients were randomly divided into a training set (n = 104) and a test set (n = 45). **Table 1** displays the statistics of clinical features. There were significant differences in tumor size, ER and HER2 status between the high Ki-67 group and the low Ki-67 group in the training set and the test set (*p* < 0.05). Additionally, PR status differed significantly in the training set (*p* < 0.05). However, no notable differences were found in age, location of mass, menstrual status, and lymph node metastasis in either the training set or the test set (*p* > 0.05).

**Table 1:** Statistical results of the clinical characteristics.

characteristics	training set		*p*	test set		*p*
	High Ki-67	Low Ki-67		High Ki-67	Low Ki-67	
	(n=48)	(n=56)		(n=19)	(n=26)	
Age, Mean ± SD	58.58 ± 10.90	59.96 ± 13.04	0.567	58.47 ± 9.54	58.15 ± 11.84	0.925
Location of the mass,n(%)			0.716			0.493
right	24(50%)	30(53.6%)		9(47.4%)	15(57.7%)	
left	24(50%)	26(46.4%)		10(52.6%)	11(42.3%)	
Menstruation status,n(%)			0.467			0.368
Yes	16(33.3%)	15(26.8%)		3(15.8%)	7(26.9%)	
No	32(66.7%)	41(73.2%)		16(84.2%)	19(73.1%)	
ER,n(%)			0.044*			0.044*
positive	30(62.5%)	48(85.7%)		10(52.6%)	21(80.8%)	
negative	18(37.5%)	12(14.3%)		9(47.4%)	5(19.2%)	
PR,n(%)			0.000*			0.135
positive	17(35.4%)	40(71.4%)		6(31.6%)	14(53.8%)	
negative	31(64.6%)	16(28.6%)		13(68.4%)	12(46.2%)	
HER2,n(%)			0.029*			0.007*
positive	18(37.5%)	33(58.9%)		10(52.6%)	4(15.4%)	
negative	30(62.5%)	23(41.1%)		9(47.4%)	22(84.1%)	
LNM, n(%)			0.242			0.912
positive	18(37.5%)	15(26.8%)		7(36.8%)	10(38.5%)	
negative	30(62.5%)	41(73.2%)		12(63.2%)	16(61.5%)	
Calcifications,n(%)			0.070			0.670
positive	21(43.7%)	15(26.8%)		7(36.8%)	8(30.8%)	
negative	27(56.3%)	41(73.2%)		12(63.2%)	18(69.2%)	
Burr edges,n(%)			0.079			0.314
positive	20(41.7%)	33(58.9%)		12(63.2%)	20(76.9%)	
negative	28(58.3%)	23(41.1%)		7(36.8%)	6(23.1%)	
Tumor size(mm), Mean ± SD	21.77 ± 5.35	17.04 ± 5.68	0.000*	22.63 ± 8.23	16.54 ± 4.81	0.003*

ER, estrogen receptor; PR, progesterone receptor; HER2, human epidermal growth factor receptor 2; SD, standard deviation; LNM, lymph node metastasis; *p<0.05.

### 3.2 Radiomics feature selection

**Figure 3:**
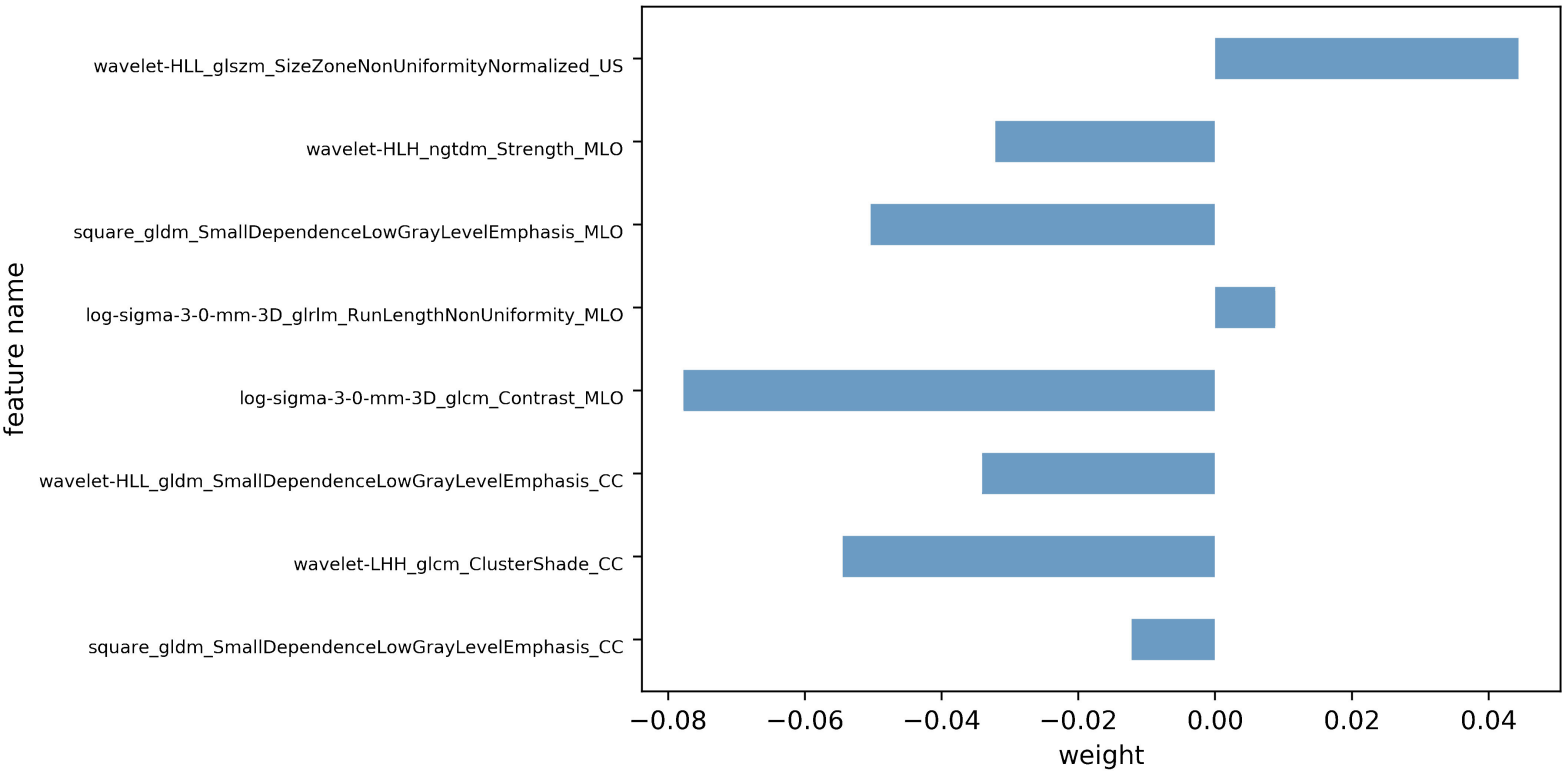
Weights of non-zero coefficient features after the LASSO algorithm.

Following feature dimensionality reduction and screening based on LASSO, 9 optimal features were selected for the DBT model and 3 for the US model (detailed in **Supplementary Tables 1, 2**). The fusion model incorporated 8 optimal features, including 1 feature from the US and 7 features from DBT (4 from the MLO view and 3 from the CC perspective) as detailed in **Table 2**. All of them were texture features after wavelet, LoG, and square transformations, mainly including gray-level co-occurrence matrix (GLCM) and gray-level dependence matrix (GLDM), gray-level size zone matrix (GLSZM), gray-level run length matrix (GLRLM), and neighboring gray-tone difference matrix (NGTDM), with AUC values vary from 0.556 to 0.783. The weight map of the fusion modal features is displayed in **Figure 3**, of which, wavelet-LHH_glcm_ClusterShade_CC, log-sigma-3-0-mm-3D_glcm_Contrast_MLO, square_gldm_SmallDependenceLowGrayLevelEmphasis_MLO and wavelet-HLL_glszm_SizeZoneNonUniformityNormalized_US were more weighted. In the high Ki-67 group, the mean values of the four features were -0.36 ± 0.59, -0.18 ± 0.79, -0.27 ± 0.75, 0.19 ± 1.22, and 0.11 ± 0.89, 0.19 ± 1.12, 0.43 ± 1.31, -0.38 ± 1.02 in low Ki-67 group (detailed in **Supplementary Figure 1**).

**Table 2:** Features for the prediction of the Ki-67 level in merge_SVM.

Radiomics feature	source	set	AUC	ACC	SPE	SEN
square_gldm_SmallDependenceLowGrayLevelEmphasis	DBT_CC	training	0.634	0.510	0.821	0.146
		test	0.621	0.622	0.923	0.211
wavelet_LHH_glcm_ClusterShade	DBT_CC	training	0.620	0.558	0.911	0.146
		test	0.556	0.698	0.923	0.053
wavelet_HLL_gldm_SmallDependenceLowGrayLevelEmphasis	DBT_CC	training	0.613	0.596	0.857	0.292
		test	0.640	0.622	0.769	0.421
log-sigma-3D_glcm_Contrast	DBT_MLO	training	0.661	0.644	0.768	0.500
		test	0.583	0.533	0.654	0.368
log-sigma-3D_glrlm_RunLengthNonUniformity	DBT_MLO	training	0.656	0.567	0.875	0.208
		test	0.761	0.711	1.000	0.316
square_gldm_SmallDependenceLowGrayLevelEmphasis	DBT_MLO	training	0.636	0.635	0.607	0.667
		test	0.660	0.667	0.654	0.684
wavelet_HLH_ngtdm_Strength	DBT_MLO	training	0.624	0.596	0.357	0.875
		test	0.783	0.622	0.842	0.842
wavelet_HLL_glszm_SizeZoneNonUniformityNormalized	US	training	0.616	0.538	0.929	0.083
		test	0.660	0.622	0.962	0.158

AUC, Area Under Curve; ACC, accuracy; SPE, specialty; SEN, sensitivity.

### 3.3 Assessment of model efficacy

**Figure 4:**
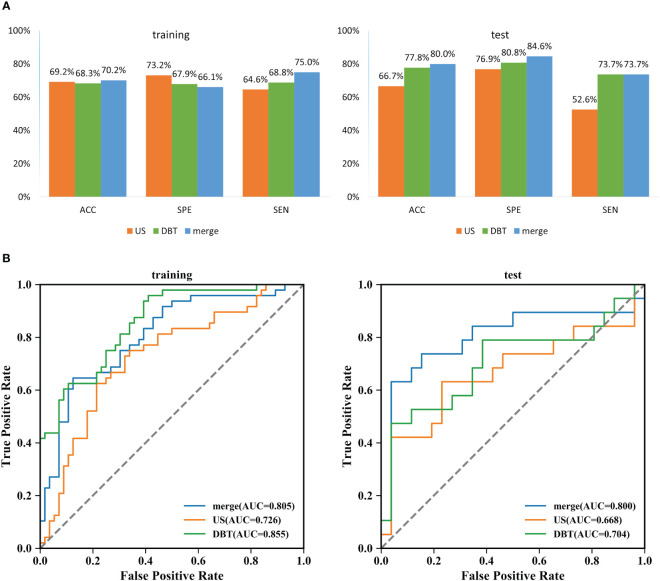
Classification results of three SVM models for predicting Ki-67 expression in breast cancer. **(A)** Comparison results of accuracy, specificity, and sensitivity of the three models. ACC: accuracy, SPE: specificity, and SEN: sensitivity. **(B)** Summary of ROC curves of the three SVM models for predicting Ki-67 expression in breast cancer.

**Figure 5:**
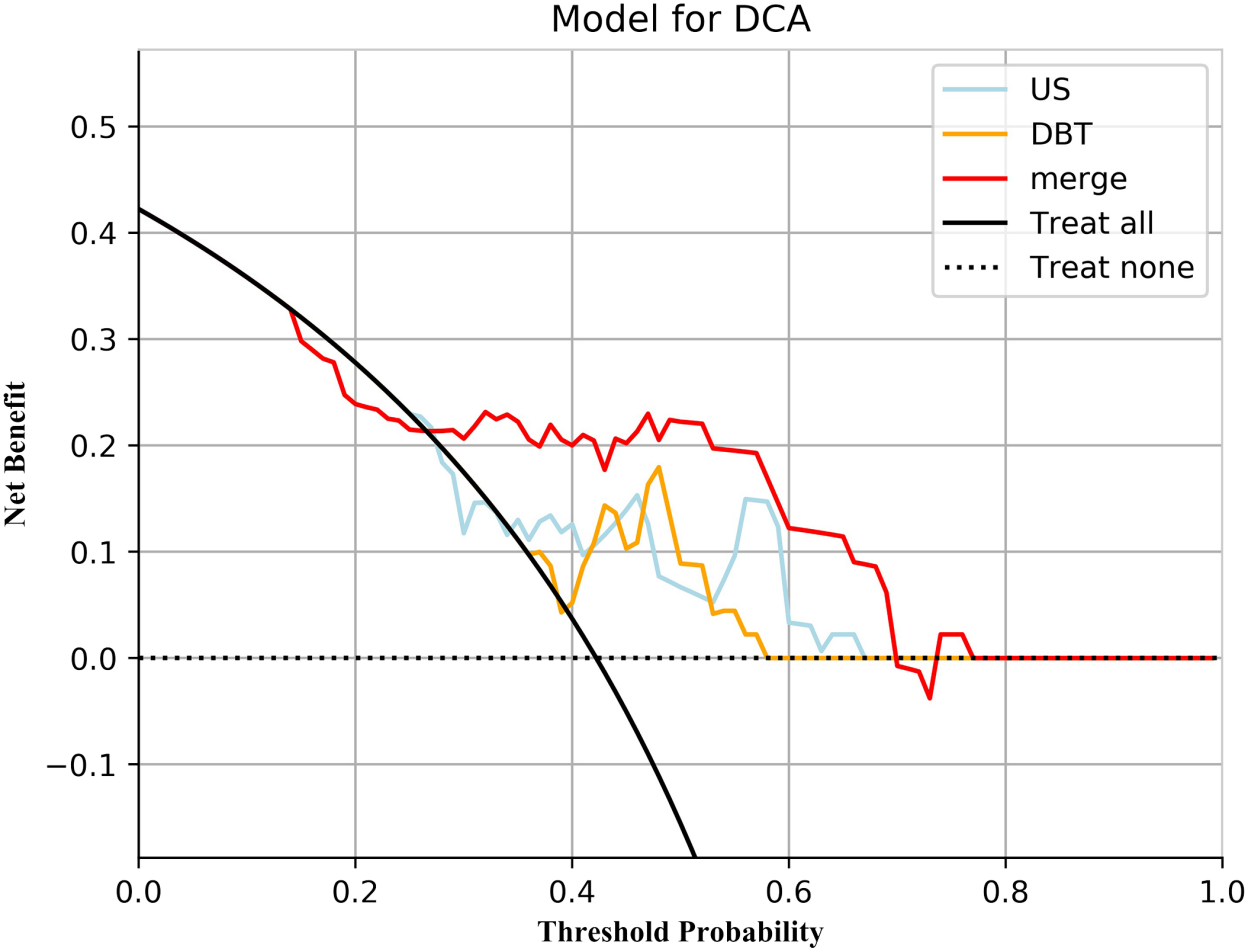
Decision curve analysis for the three models. Neither treatment as the baseline (black dashed line), merge_SVM had the highest net benefit compared to DBT_SVM, US_SVM, and both treatments (black solid line). The net benefit refers to the proportion of patients who can be treated more who are truly Ki-67 high-expressing without increasing overtreatment, compared to neither treatment, using a model or criteria to guide whether to treat or not.

The prediction performance evaluation results for the three models are displayed in **Table 3**. After comparing the performance of the three classifiers, it was found that the RF classifier was generally less effective, LR had the best prediction results in the test set but poor classification results in the training set, and SVM showed stable performance in both sets. Therefore, SVM was chosen as the preferred method for modeling due to its comprehensive performance. **Figure 4** displays the classification outcomes of the three models constructed by the SVM classifier. In the training set, the AUC values for US_SVM and DBT_SVM were 0.726 (95% CI: 0.627-0.825) and 0.856 (95% CI: 0.780-0.931) respectively. In the test set, the AUC values were 0.668 (95% CI: 0.505-0.832) for US_SVM and 0.704 (95% CI: 0.546-0.863) for DBT_SVM. Overall, DBT_SVM had a slightly higher prediction accuracy than US_SVM. The AUC of the merge_SVM model achieved 0.805 (95% CI: 0.719-0.891) in the training group and 0.800 (95% CI: 0.662-0.937) in the validation group. It had the highest AUC value in the test set and the highest accuracy, specificity, and sensitivity in both training and test sets, except for a slightly lower specificity in the training set. The DeLong test indicated that the AUC value of the merge_SVM was significantly different from the US_SVM (*p* = 0.048) and not substantially different from the DBT_SVM (*p* = 0.149). The DCA of merge_SVM expressed the best among the three models, indicating that this model had a higher net clinical benefit than the other two models for predicting high Ki-67 expression (**Figure 5**).

**Table 3:** Prediction performance of three classifiers.

Modality	training set				test set			
	AUC (95%CI)	ACC	SPE	SEN	AUC (95%CI)	ACC	SPE	SEN
SVM								
US	0.726 (0.627- 0.825)	0.692	0.732	0.646	0.668 (0.505- 0.832)	0.667	0.769	0.526
DBT	0.856 (0.780- 0.931)	0.683	0.679	0.688	0.704 (0.546- 0.863)	0.778	0.808	0.737
merge	0.805 (0.719- 0.891)	0.702	0.661	0.750	0.800 (0.662- 0.937)	0.800	0.846	0.737
RF								
US	0.788 (0.699- 0.877)	0.702	0.732	0.667	0.652 (0.486- 0.817)	0.644	0.731	0.526
DBT	0.747 (0.652- 0.843)	0.712	0.714	0.833	0.692 (0.532- 0.852)	0.622	0.692	0.526
merge	0.762 (0.668- 0.856)	0.635	0.732	0.521	0.757 (0.609- 0.905)	0.778	0.923	0.579
LR								
US	0.681 (0.577- 0.785)	0.663	0.732	0.583	0.741 (0.590- 0.892)	0.756	0.808	0.684
DBT	0.763 (0.670- 0.856)	0.702	0.679	0.708	0.812 (0.678- 0.945)	0.756	0.769	0.737
merge	0.777 (0.686- 0.868)	0.683	0.643	0.729	0.816 (0.683- 0.948)	0.822	0.885	0.737

SVM, Support Vector Machine; RF, Random Forest; LR, Logistic Regression; AUC, Area Under Curve; ACC, accuracy; SPE, specialty; SEN, sensitivity.

### 3.4 Assessment of the nomogram

**Figure 6:**
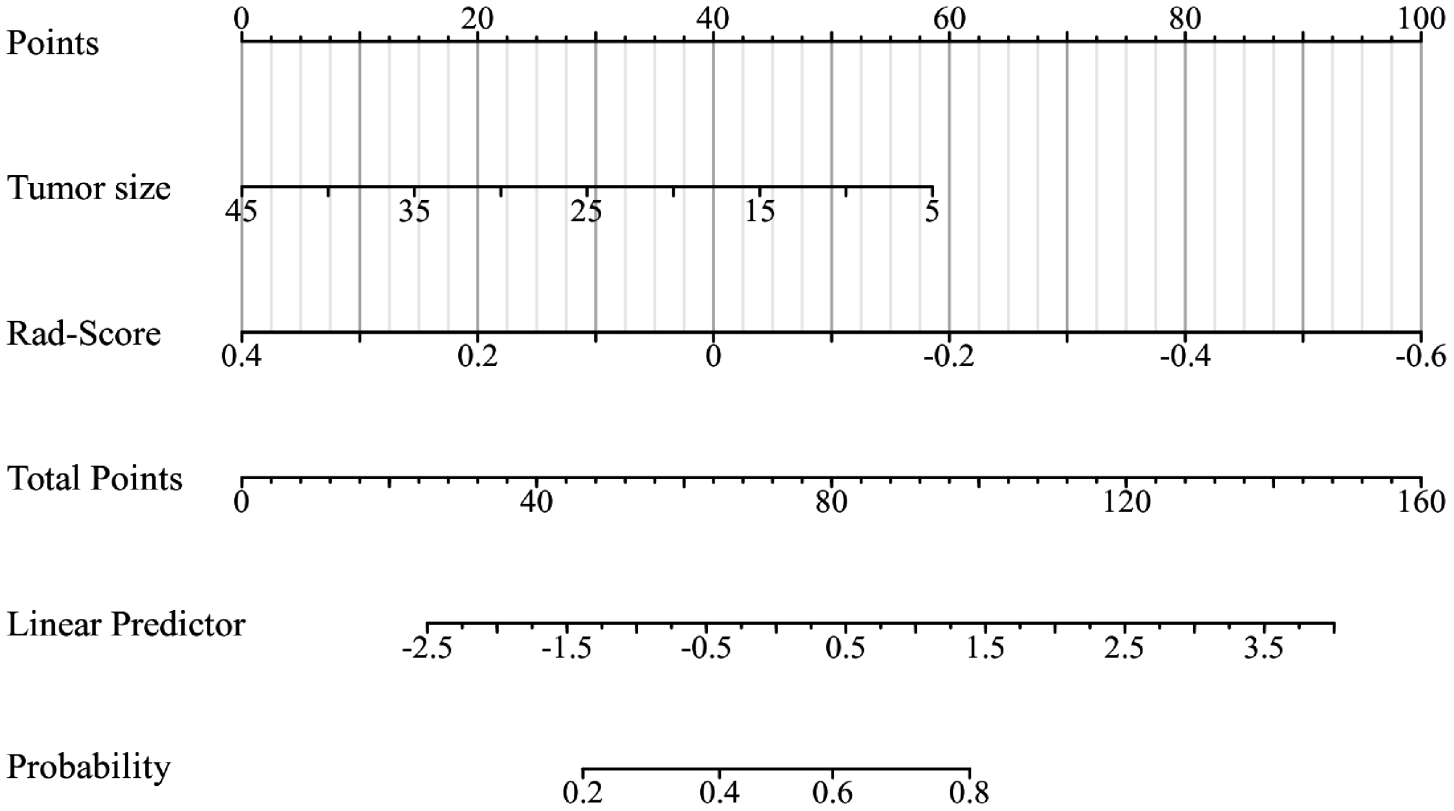
The radiomics-clinical nomogram was developed by logistic regression, which includes tumor size and Rad-Score.

**Figure 7:**
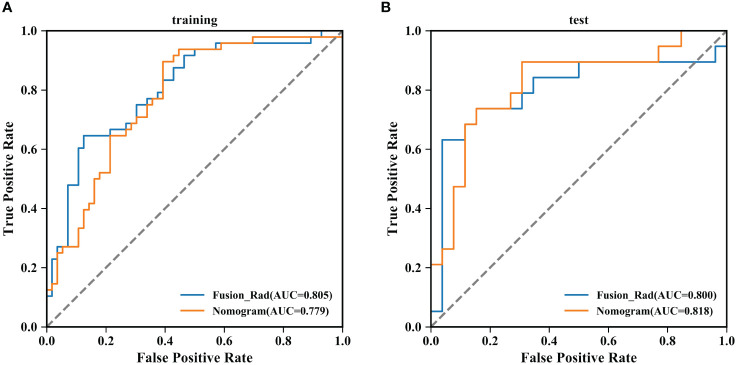
Summary of ROC curves of the Fusion-Rad and Nomogram models for predicting Ki-67 expression in breast cancer. **(A)** training set; **(B)** test set.

**Figure 8:**
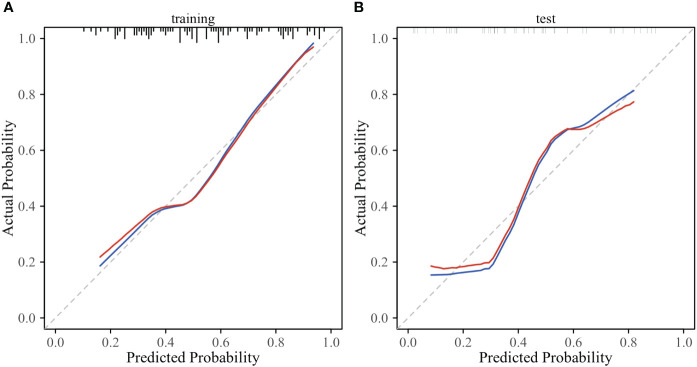
Calibration curves of the nomogram model in the training **(A)** and test sets **(B)**.

**Figure 9:**
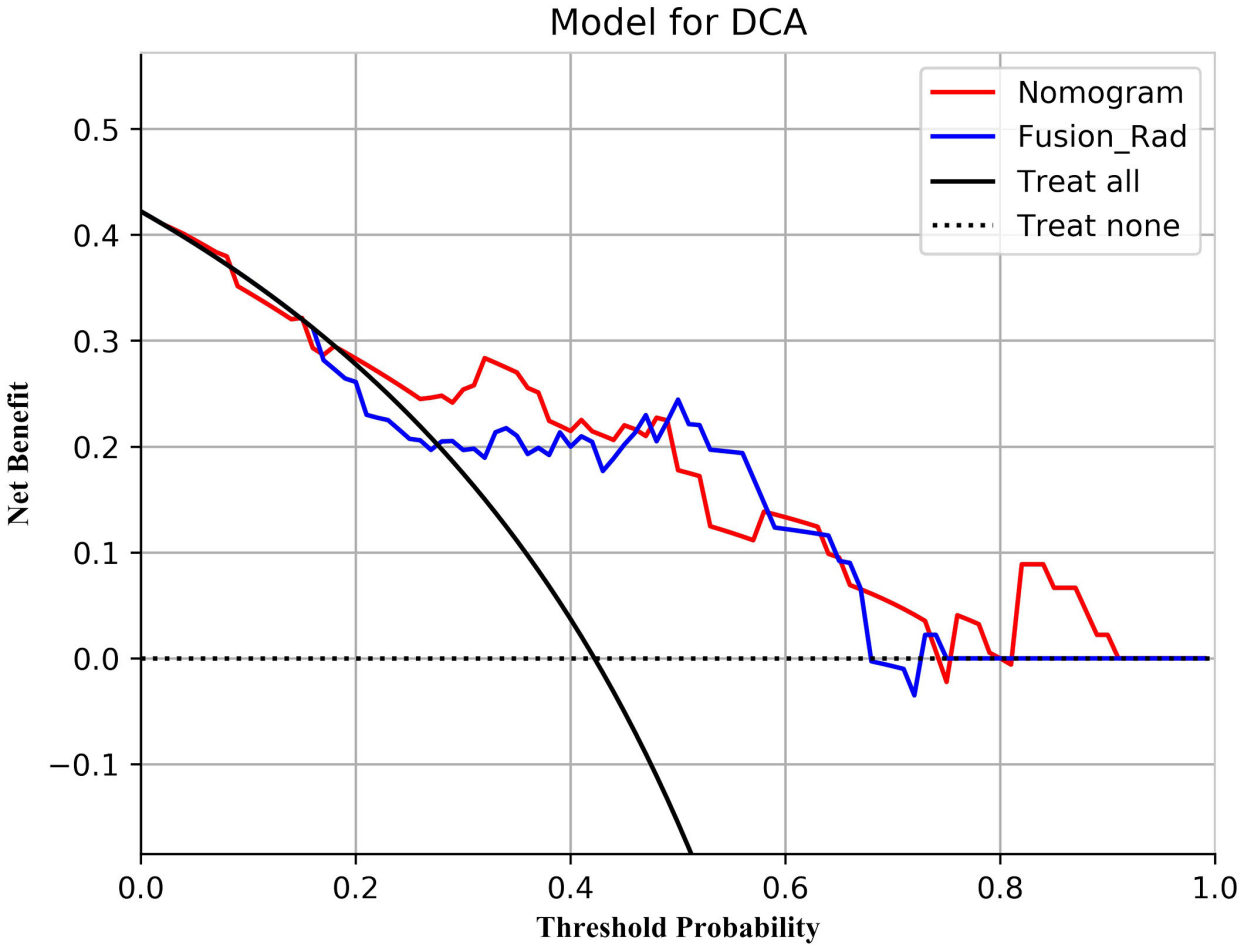
Decision curve analysis for the Fusion-Rad and Nomogram models in predicting Ki-67 status for breast cancer patients.

According to the performance of the three radiomics models above, merge_SVM was selected as the Rad-Score model (Fusion-Rad model), then established a rad score formula to calculated each patient’s Rad-Score using non-zero features coefficient. Rad-score and clinical characteristics were analyzed utilizing the univariate and multivariate logistic regression, as shown in **Table 4**. Among them, tumor size and Rad-Score were relevant to Ki-67 expression level (*p* < 0.001). In multivariate logistic regression, tumor size (OR 0.925, CI 0.849 - 1.002, *p* < 0.05), and Rad-Score (OR 0.006, CI -3.174-3.186, *p* < 0.05) were independent risk factors for Ki-67 status in BC. A nomogram was developed by integrating Rad-Score, tumor size using the logistic regression method (**Figure 6**). The AUC values of the nomogram models were 0.779 in the training set and 0.818 in the test set (**Figure 7**, **Table 5**). The calibration curves revealed good predictive accuracy between model-predicted values and actual outcomes (**Figure 8**). The DCA of the nomogram model performed better than the Fusion-Rad model (**Figure 9**).

**Table 4:** Univariate and multivariate logistic regression results of the risk factors of high Ki-67 level in BC.

Characteristics	Univariate analysis		Multivariate analysis	
	OR (95% CI)	*p*	OR (95% CI)	*p*
Age	1.003 (0.976 - 1.031)	0.807		
Tumor size	0.855 (0.788 - 0.922)	< 0.001*	0.925 (0.849 - 1.002)	0.046*
Calcifications	1.842 (1.158 - 2.526)	0.080	1.403 (0.627 - 2.179)	0.392
Menstruation status	1.015 (0.298 - 1.733)	0.967		
Location of the mass	0.798 (0.151 - 1.445)	0.494		
Rad-Score	0.001 (-2.788 - 2.790)	< 0.001*	0.006 (-3.174 - 3.186)	0.002*

*p<0.05.

**Table 5:** Performance of predicting Ki-67 levels in different models of training groups and validation groups.

Model	AUC (95% CI)
	Training group
Fusion-Rad model	0.805 (0.719-0.891)
Nomogram model	0.779 (0.690-0.868)
	Validation group
Fusion-Rad model	0.800 (0.662-0.937)
Nomogram model	0.818 (0.685-0.950)

## 4 Discussion

Early prediction of Ki-67 expression level in breast cancer has important clinical significance for breast cancer treatment planning and prognosis (21). Currently, the detection of Ki-67 is mainly based on IHC, which is an invasive procedure that is time-consuming and non-repeatable. A non-invasive preoperative detection method is needed in clinical practice. Multiple studies have proved that radiomics techniques can be used for the prediction of therapy response and prognosis (22), and tumor biomarkers such as Ki-67 (23). In this research, we extracted an amount quantity of radiomics features from DBT and US images, and then constructed and validated the radiomics predictive models of Ki-67 expression levels in unimodal and fusion modalities. Compared with the DBT and US models, the fusion model achieved better classification results in the test set (AUC=0.800). The DCA indicated that the net clinical benefit of merge_SVM was higher than that of DBT_SVM and US_SVM, indicating that the fusion model has higher clinical applicability and can provide a non-invasive prediction method for Ki-67 level in breast cancer.

In this study, we first established prediction models for Ki-67 expression levels in BC based on two clinically common imaging modalities, DBT and US. Our DBT model has an AUC value of 0.704, which is similar to the performance of Tagliafico’s model (AUC = 0.698) (24). Similarly, our US model has an AUC value of 0.668, which is not much different from the results of the US model constructed by Liu et al. (AUC = 0.713) (25). It is essential to mention that the AUC value of the DBT model in this study was 0.036 higher than that of the US. Additionally, the accuracy, specificity, and sensitivity were also higher. This difference may be related to the different imaging modalities used in DBT and US. DBT utilizes three-dimensional imaging, allowing for the capture of crucial details like microcalcification clusters, burr edges, and architectural distortion of the tumor (9). In contrast, 2D US images provide limited information due to the limitation of ultrasound scanning probes. However, the predictive ability of unimodal models is always limited, and it has been suggested that combining multiple imaging modalities can improve the accuracy of Ki-67 prediction (26, 27). The results of this study showed that the fusion model constructed based on US and DBT has an AUC value of 0.800 (accuracy of 80.0%), surpassing the unimodal models in classification. This indicates that the combination of DBT and US can compensate for the shortcomings of the imaging modalities, provide more heterogeneous tumor information, improving the prediction level. It is worth mentioning that the prediction performance of the fusion model constructed in this study for Ki-67 expression level is comparable to that of the MRI multimodal models. Jiang et al. (26) combined the radiomics features of two MRI modalities, dynamic contrast-enhanced (DCE) and diffusion-weighted (DW), and the predicted AUC for Ki-67 status was 0.818. Fan et al. (28) combined precontrast and apparent diffusion coefficient images, the predicted AUC for Ki-67 achieved 0.811. Our results are consistent with the conclusions of a recent meta-analysis (17), that combining DBT and US can enhance the diagnosis accuracy of dense breasts and potentially serve as an alternative to MRI.

The fusion model was effective in enhancing the estimated performance of Ki-67, however, the gain was limited. When comparing the prediction performance of the fusion model with unimodal models, the AUC value of the fusion model improves significantly compared to the US model (DeLong *p* < 0.05), but shows only a slight improvement compared to the DBT model (DeLong *p* > 0.05). The reason for such a result may be due to the deviation in the proportion of the two unimodal feature numbers incorporated in the fusion model, with 7 DBT image features but only 1 US image feature. Additionally, DBT contains images at both CC and MLO views, doubling the number of features and providing more tumor information. Therefore, this study concluded that the fusion model could enhance the accuracy of predicting Ki-67. However, if resources are limited and patients cannot take both imaging examinations at the same time, it is preferable to choose DBT as a preoperative assessment modality.

In this study, the eight radiomics features incorporated in the fusion model were all transformed higher-order texture features, including 2 GLCM, 3 GLDM, 1 GLRLM, 1 GLSZM, and 1 NGTDM features. These features can characterize and quantify the texture attributes and complexity in the whole region, suggesting that texture complexity is associated with the Ki-67 status of breast cancer, which can serve as an important predictor of Ki-67 expression levels. This discovery is similar to previous research that has emphasized the importance of texture features (24, 29–31). The GLCM features quantify the complexity of voxel intensities in the tumor region (32). Among GLCM features, Cluster Shade is a measure of skewness and homogeneity. Li et al. (33) demonstrated that the Cluster Shade value was higher in the cervical cancer Ki-67 high expression group compared to the low expression group. In this study, the absolute value of GLCM_ClusterShade was higher in the Ki-67 high expression group than the low expression group, indicating that the breast cancer Ki-67 high expression group may have rougher image texture, higher tumor heterogeneity, and more invasiveness. The GLDM features indicate the degree of roughness of the image texture (32). Petrillo et al. (34) found that these features are effective in distinguishing between benign and malignant breast lesions and in identifying the HER2 status. In the present study, three GLDM features showed better performance in classifying Ki-67 levels (with accuracies of 62.2%, 62.2%, and 66.7%, respectively), indicating that GLDM also can identify Ki-67 status. Texture features in US images can be applied to predict Ki-67 status (25, 31) and lymph node metastasis in breast carcinoma (35, 36). Previous research focused on the importance of GLCM, while the present study found that the GLSZM features in US images also have significant predictive value (accuracy of 66.0%). The GLSZM features quantify the grey-scale changes of the connected regions at the edge of the image, reflecting the clarity of the tumor edge. Niu et al. (37) found that the GLSZM values of benign lesions were higher than that of malignant lesions, which proved that the edges of benign lesions were clearer than those of malignant ones. This study found that the absolute value of the GLSZM features was higher in the low Ki-67 group compared to the high Ki-67 group, suggesting that the tumor margins may be more distinct in BC patients with low Ki-67 expression level.

In this study, ER and HER2 status had intergroup differences (*p* < 0.05), which suggests that Ki-67 values may be higher in patients with ER and HER2 positivity. According to the results of the univariate and multivariate logistic regression, the size of tumors and Rad-Score could be considered as independent risk factors to predict high Ki-67 level in BC. In order to facilitate the potential utilization of radiomics methods by clinical doctors, we developed a nomogram model incorporating Rad-Score and clinical independent predictors (tumor size), which achieved the best predictive performance (AUC=0.818). The DCA shows that the nomogram has better clinical applicability as a predictor of Ki-67 expression. To utilize our nomogram, clinicians should delineate the ROIs in DBT and US images to acquire Rad-Score, then calculate the probability of high Ki-67 status via the Rad-Score and the value of tumor size. Afterwards, clinicians can combine these probabilities with the patient’s other clinical characteristics to make a comprehensive assessment.

There exist some limitations. (a) This research is a single-center retrospective study with a small sample capacity, which makes it difficult to avoid selective bias. To confirm the accuracy of the models, it is necessary to have more cases of illness and multi-center data. (b) The US features were extracted from s single image of the largest cross-section of the tumor, which may result in the missing of crucial information about tumor heterogeneity. (c) The features we obtained after the screening were all texture-related features, ignoring features such as shape features and first-order features, probably because the difference in those features between the two groups was small or the weight of the features was low. In future research, we will improve the feature selection method to explore the clinical significance of these features. (d) Manual outlining of the ROI not only increases the time needed but also the inconsistency between different radiologists. In future research, deep learning will be explored as a means to segment the ROIs to substitute manual outlining automatically. Additionally, the potential of deep learning to enhance the accuracy of classification will be investigated.

In conclusion, the fusion model developed based on radiomics features of DBT and US images is superior to the unimodal models, which might assist in predicting the Ki-67 expression level of BC patients and provide individualized precision treatment. However, the gain effect of the fusion model is limited, so it is recommended that DBT be preferred as a preoperative diagnostic modality when resources are restricted. Otherwise, the nomogram offers predictive advantages over other methods and can be a valuable tool for predicting Ki-67 levels in breast cancer.

## Data availability statement

The raw data supporting the conclusions of this article will be made available by the authors, without undue reservation.

## Ethics statement

The studies involving humans were approved by Ethics Committee of the Nanjing Medical University Affiliated Suzhou Hospital. The studies were conducted in accordance with the local legislation and institutional requirements. Written informed consent for participation was not required from the participants or the participants’ legal guardians/next of kin in accordance with the national legislation and institutional requirements.

## Author contributions

JL: Conceptualization, Data curation, Formal analysis, Investigation, Methodology, Software, Validation, Writing – original draft, Writing – review & editing. CY: Data curation, Formal analysis, Investigation, Methodology, Software, Validation, Writing – original draft, Writing – review & editing. JG: Conceptualization, Resources, Supervision, Writing – review & editing. CL: Data curation, Formal analysis, Supervision, Validation, Writing – original draft. YW: Data curation, Formal analysis, Validation, Writing – original draft. QC: Data curation, Formal analysis, Validation, Writing – original draft. YC: Funding acquisition, Supervision, Writing – review & editing. SC: Conceptualization, Funding acquisition, Resources, Supervision, Writing – original draft, Writing – review & editing.
