# Supplementary material for: Predicting Ki-67 expression levels in breast cancer using radiomics-based approaches on digital breast tomosynthesis and ultrasound
Source: Front Oncol. 2024 Jul 11;14:1403522. doi: 10.3389/fonc.2024.1403522 (PMC11269194; doi:10.3389/fonc.2024.1403522)
Supplement: Supplementary file 1 [file Table_1.docx]

## Table 2 Features for the prediction of the Ki-67 level in DBT_SVM

| Radiomics feature | source | set | AUC | ACC | SPE | SEN |
| --- | --- | --- | --- | --- | --- | --- |
| square_gldm_SmallDependenceLowGrayLevelEmphasis | DBT_CC | training | 0.617 | 0.567 | 0.482 | 0.667 |
|  |  | test | 0.384 | 0.422 | 0.462 | 0.368 |
| wavelet-LHH_glcm_ClusterShade | DBT_CC | training | 0.619 | 0.615 | 0.768 | 0.438 |
|  |  | test | 0.698 | 0.622 | 0.769 | 0.421 |
| wavelet-HLL_gldm_SmallDependenceLowGrayLevelEmphasis | DBT_CC | training | 0.613 | 0.596 | 0.750 | 0.417 |
|  |  | test | 0.640 | 0.578 | 0.692 | 0.421 |
| log-sigma-1-0-mm-3D_glcm_Contrast | DBT_MLO | training | 0.585 | 0.558 | 0.535 | 0.583 |
|  |  | test | 0.711 | 0.644 | 0.577 | 0.737 |
| log-sigma-3-0-mm-3D_glcm_Contrast | DBT_MLO | training | 0.658 | 0.615 | 0.696 | 0.521 |
|  |  | test | 0.577 | 0.556 | 0.696 | 0.474 |
| log-sigma-3-0-mm-3D_glrlm_RunVariance | DBT_MLO | training | 0.717 | 0.625 | 0.732 | 0.500 |
|  |  | test | 0.670 | 0.667 | 0.846 | 0.421 |
| log-sigma-3-0-mm-3D_glrlm_RunLengthNonUniformity | DBT_MLO | training | 0.656 | 0.567 | 0.821 | 0.271 |
|  |  | test | 0.761 | 0.667 | 0.923 | 0.316 |
| square_gldm_SmallDependenceLowGrayLevelEmphasis | DBT_MLO | training | 0.634 | 0.625 | 0.625 | 0.625 |
|  |  | test | 0.654 | 0.644 | 0.692 | 0.579 |
| wavelet-HLH_ngtdm_Strength | DBT_MLO | training | 0.624 | 0.625 | 0.429 | 0.854 |
|  |  | test | 0.783 | 0.622 | 0.462 | 0.842 |
| AUC: Area Under Curve; ACC: accuracy; SPE: specialty; SEN: sensitivity. | | | | | | |
