# Supplementary material for: Predicting Ki-67 expression levels in breast cancer using radiomics-based approaches on digital breast tomosynthesis and ultrasound
Source: Front Oncol. 2024 Jul 11;14:1403522. doi: 10.3389/fonc.2024.1403522 (PMC11269194; doi:10.3389/fonc.2024.1403522)
Supplement: Supplementary file 2 [file Table_2.docx]

## Table 1 Features for the prediction of the Ki-67 level in US_SVM

| Radiomics feature | source | set | AUC | ACC | SPE | SEN |
| --- | --- | --- | --- | --- | --- | --- |
| logarithm_glrlm_GrayLevelNonUniformity | US | training | 0.652 | 0.596 | 0.696 | 0.479 |
|  |  | test | 0.719 | 0.689 | 0.769 | 0.579 |
| wavelet-LLH_glrlm_ShortRunHighGrayLevelEmphasis | US | training | 0.662 | 0.596 | 0.696 | 0.479 |
|  |  | test | 0.708 | 0.667 | 0.808 | 0.474 |
| wavelet-HLL_glszm_SizeZoneNonUniformityNormalized | US | training | 0.617 | 0.567 | 0.500 | 0.646 |
|  |  | test | 0.660 | 0.689 | 0.692 | 0.684 |
| AUC: Area Under Curve; ACC: accuracy; SPE: specialty; SEN: sensitivity. | | | | | | |
