# Supplementary material for: Predicting Ki-67 expression levels in breast cancer using radiomics-based approaches on digital breast tomosynthesis and ultrasound
Source: Front Oncol. 2024 Jul 11;14:1403522. doi: 10.3389/fonc.2024.1403522 (PMC11269194; doi:10.3389/fonc.2024.1403522)
Supplement: Supplementary file 3 [file Table_3.docx]

Rad-Score=

(-0.012221) *square_gldm_SmallDependenceLowGrayLevelEmphasis_CC

+(-0.054481) *wavelet-LHH_glcm_ClusterShade_CC

+(-0.034094) *wavelet-HLL_gldm_SmallDependenceLowGrayLevelEmphasis_CC

+(-0.077736) *log-sigma-3-0-mm-3D_glcm_Contrast_MLO

+(0.008803) *log-sigma-3-0-mm-3D_glrlm_RunLengthNonUniformity_MLO

+(-0.050399) *square_gldm_SmallDependenceLowGrayLevelEmphasis_MLO

+(-0.032149) *wavelet-HLH_ngtdm_Strength_MLO

+(0.044396) *wavelet-HLL_glszm_SizeZoneNonUniformityNormalized_US
