# Supplementary figures and images for: Predicting Ki-67 expression levels in breast cancer using radiomics-based approaches on digital breast tomosynthesis and ultrasound
Source: Front Oncol. 2024 Jul 11;14:1403522. doi: 10.3389/fonc.2024.1403522 (PMC11269194; doi:10.3389/fonc.2024.1403522)

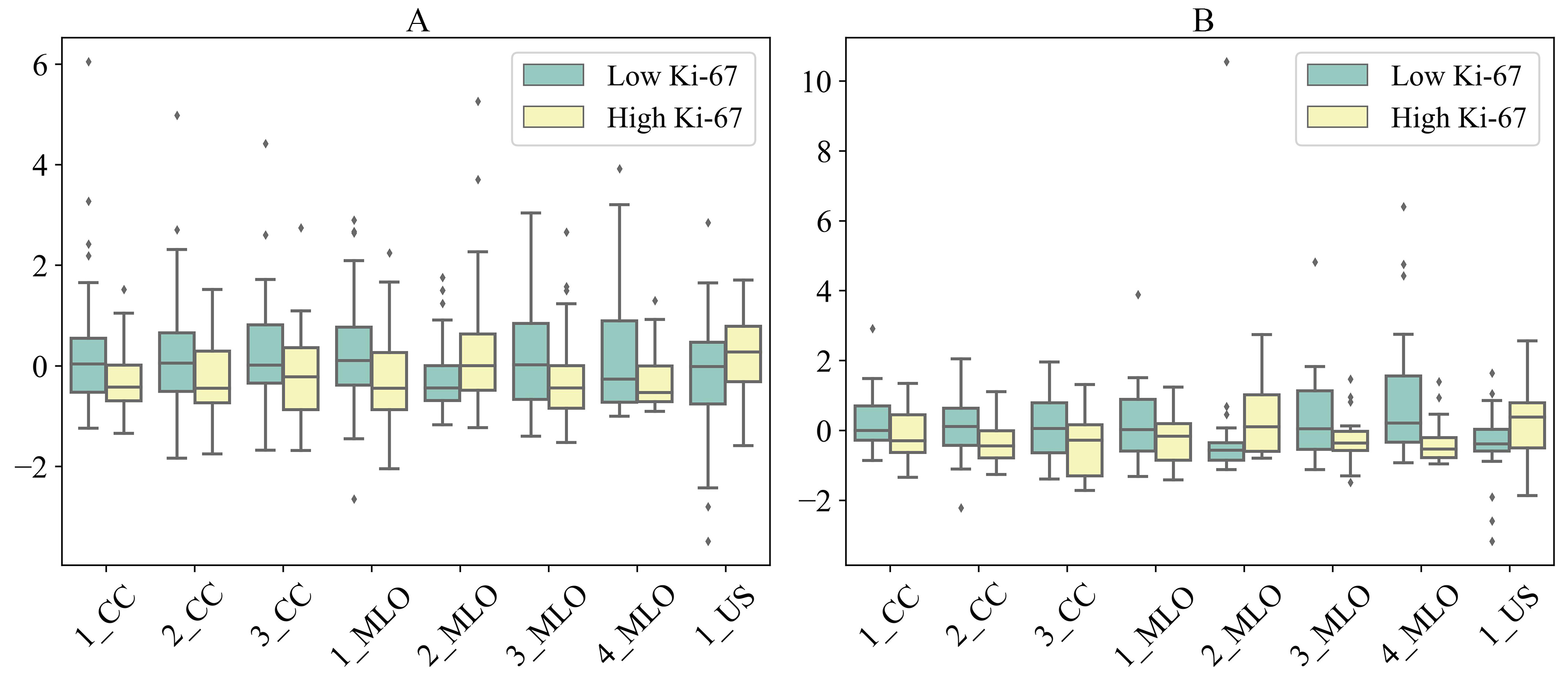

Supplement: Supplementary file 4 [file Image_1.tif]
